# Comprehensive Immune Repertoires of T Cell Receptor and B Cell Receptor Identify Key Immune Features Associated With Disease Activity of Systemic Lupus Erythematosus

**DOI:** 10.1002/mco2.71087

**Published:** 2026-10-23

**Authors:** Jingyi Wu, Zhuochao Zhou, Anrui Yu, Xia Lin, Yuyang Ren, Jialin Teng, Dehao Zhu, Fan Wang, Yijun You, Xiangjun Ji, Puyu Wang, Hongpu Li, Chengde Yang, Wenbo Wang, Yawei Du, Junna Ye

**Affiliations:** ^1^ Department of Rheumatology and Immunology Ruijin Hospital Shanghai Jiao Tong University School of Medicine Shanghai China; ^2^ Department of Paediatrics Ruijin Hospital Shanghai Jiao Tong University School of Medicine Shanghai China; ^3^ Department of Critical Care Medicine Ruijin Hospital Shanghai Jiao Tong University School of Medicine Shanghai China; ^4^ Department of Infectious Diseases Ruijin Hospital Shanghai Jiao Tong University School of Medicine Shanghai China; ^5^ Department of Oncology Shanghai Tenth People's Hospital Tongji University School of Medicine Shanghai China; ^6^ LeaLing Biopharma Co., Ltd. Suzhou China; ^7^ Department of Rheumatology and Immunology The Fourth Affiliated Hospital of Xinjiang Medical University Xinjiang China; ^8^ Department of Orthopaedics Shanghai Key Laboratory for Prevention and Treatment of Bone and Joint Diseases Shanghai Institute of Traumatology and Orthopaedics Ruijin Hospital Shanghai Jiao Tong University School of Medicine Shanghai China

**Keywords:** BCR, disease activity, immune repertoire sequencing, systemic lupus erythematosus, TCR

## Abstract

We integrated immune repertoire sequencing (CD4+ and CD8+ T cell receptor [TCR], B cell receptor [BCR]) and transcriptional profiling to characterize the peripheral immune landscape in 32 active systemic lupus erythematosus (SLE) patients, 30 stable SLE patients, and 35 healthy donors. CD4+ and CD8+ TCR α‐ and β‐chain repertoires in SLE patients showed lower diversity, reduced similarity, and more heterogeneous V–J usage, with CD8+ TCR profiles exhibiting relatively consistent stepwise changes across healthy, stable, and active groups. CD8+ T‐cell transcriptomics revealed activity‐associated upregulation of *CD38*, *S100A11*, *IFITM3*, and *LAG3*. In addition, BCR immunoglobulin heavy chain (IGH) repertoire analysis showed isotype‐specific remodeling in SLE, characterized by contracted IgM/IgD repertoires, increased IgA isotype ratio and richness, and enhanced IgG diversity. Active SLE showed further increased IgG richness with rare‐clone enrichment and higher IgM diversity. Among the TCR, BCR, and CD8+ T‐cell transcriptomic features evaluated, IGH‐derived metrics, particularly IgG‐ and IgA‐associated features, showed apparent within‐cohort discrimination between active and stable SLE. Genetic algorithm‐based label‐guided feature selection followed by hierarchical clustering revealed a multi‐omics pattern associated with disease activity within the current cohort. Our study characterized peripheral immune repertoire and transcriptional alterations in SLE and highlighted their value for characterizing disease activity.

## Graphical abstract

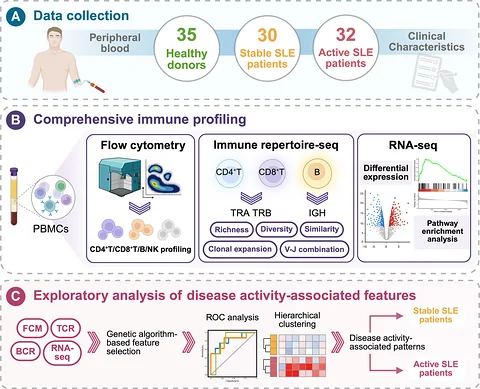

## 1 Introduction

Systemic lupus erythematosus (SLE) is an autoimmune disorder characterized by excessive autoantibody production, multiorgan involvement, and a relapsing‐remitting pattern of disease activity [1]. Fluctuations in disease activity remain a major challenge in clinical management, as active disease states are associated with organ damage and adverse long‐term outcomes [2]. At the center of this dynamic process lies a profoundly disturbed adaptive immune system, in which autoreactive T and B cells cooperate in the loss of self‐tolerance and sustain autoimmune inflammation [3]. Furthermore, biological therapies targeting B cells and T cells, including belimumab [4], rituximab [5], CAR‐T (chimeric antigen receptor‐T) [6, 7], and recent T‐cell engager therapies (TCEs) [8], are expanding the possibilities for treating refractory autoimmune disease. These advances underscore the clinical relevance of T‐ and B‐cell dysregulation in SLE, but also raise the need for a more detailed understanding of how adaptive immune states are remodeled across different levels of disease activity.

Immune repertoire, primarily encompassing T cell receptor (TCR) and B cell receptor (BCR) repertoires, provides a molecular method to examine adaptive immune remodeling [9]. TCR diversity is generated primarily through V(D)J recombination, junctional diversification, and αβ chain pairing, whereas BCR diversity is further shaped by somatic hypermutation (SHM) and class‐switch recombination (CSR) following antigen exposure [10]. Repertoire features such as diversity, clonality, complementarity‐determining region 3 (CDR3) characteristics, V(D)J usage, and immunoglobulin isotype composition may capture the imprint of antigen‐driven selection, clonal expansion, and immune activation [10, 11].

Immune repertoire profiling has revealed broad TCR and BCR abnormalities in SLE. TCR studies have reported reduced repertoire diversity, increased clonal expansion, and biased V/J gene usage, suggesting expansion of selected T‐cell clones [12, 13, 14, 15, 16, 17, 18, 19]. BCR analyses have identified enhanced clonal expansion, skewed IGHV gene usage, subset‐dependent CDR3 alterations, and altered isotype composition [17, 20, 21, 22, 23]. Among these BCR features, increased IGHV4 family usage, particularly autoreactivity‐associated IGHV4‐34 [21, 22], together with IgA overrepresentation and increased IgE switching [20], points to abnormal antigen‐driven selection and B‐cell maturation in SLE. Building on these observations, disease‐associated TCR and BCR signatures [12, 16, 18, 21, 22, 24, 25] have been explored as potential biomarkers for distinguishing SLE patients from healthy controls.

However, immune repertoire features mainly describe clonal architecture and receptor sequence composition, but may not fully capture functional T‐cell states. Transcriptomic profiling provides a complementary approach in this regard. CD8+ T cells are increasingly implicated in SLE pathogenesis, activity, and tissue injury, making them a relevant compartment for such analysis. Clonally expanded CD8+T cells have been implicated in SLE pathogenesis and SLE‐affected tissues [26]. Peripheral activated CD8+ T cells have been associated with SLE disease activity and treatment resistance [27, 28, 29], consistent with a report linking intrarenal GZMK+ CD8+ T‐cell infiltration to lupus nephritis activity and severity [30]. Together, these findings provide a rationale for profiling CD8+ T‐cell transcriptional states alongside TCR and BCR repertoire features.

Despite these advances, how immune repertoire remodeling and transcriptomic immune states relate to SLE disease activity remains incompletely defined, with existing studies yielding inconsistent conclusions [23, 31]. Moreover, TCR and BCR repertoires and transcriptomic analysis are often analyzed separately, making it difficult to determine how these immune features jointly relate to disease activity. These limitations highlight the need for simultaneous profiling and comparison of TCR, BCR, and transcriptomic features in active and stable SLE.

To address these questions, we integrated flow cytometry (FCM), CD4+ and CD8+ TCRα/β sequencing, BCR immunoglobulin heavy chain (IGH) sequencing, and CD8+ T‐cell RNA sequencing in peripheral blood from active SLE patients, stable SLE patients, and healthy donors (HDs). We applied fine‐grained repertoire metrics to capture SLE‐specific immune repertoire alterations, activity‐associated patterns of TCR‐, BCR‐, and transcriptome‐derived features, and integrated these multilayered features using a genetic algorithm‐based label‐guided feature selection followed by hierarchical clustering. This framework allowed us to characterize repertoire abnormalities in SLE and explore coordinated immune features associated with active and stable disease.

## 2 Results

### 2.1 A Comprehensive Clinical Immunoprofiling Platform of SLE Patients

In this study, we combined FCM, immune repertoire sequencing, and bulk RNA sequencing to evaluate the systemic immune characteristics of patients with SLE (Figure 1). The clinical characteristics of 32 active, 30 stable SLE patients and 35 HDs involved in this study are shown in Table 1. Background treatments at the time of sampling are summarized in Table 2. To evaluate potential treatment‐related confounding, we compared several representative treatment variables between the stable and active SLE groups. No significant differences were observed in glucocorticoid dose (*p* = 0.5771), hydroxychloroquine use (*p* = 0.302), mycophenolate mofetil use (*p* = 0.7459), or tacrolimus use (*p* = 0.418). These findings suggest that the major background treatments recorded in this cohort were not markedly different between groups.

**FIGURE 1:**
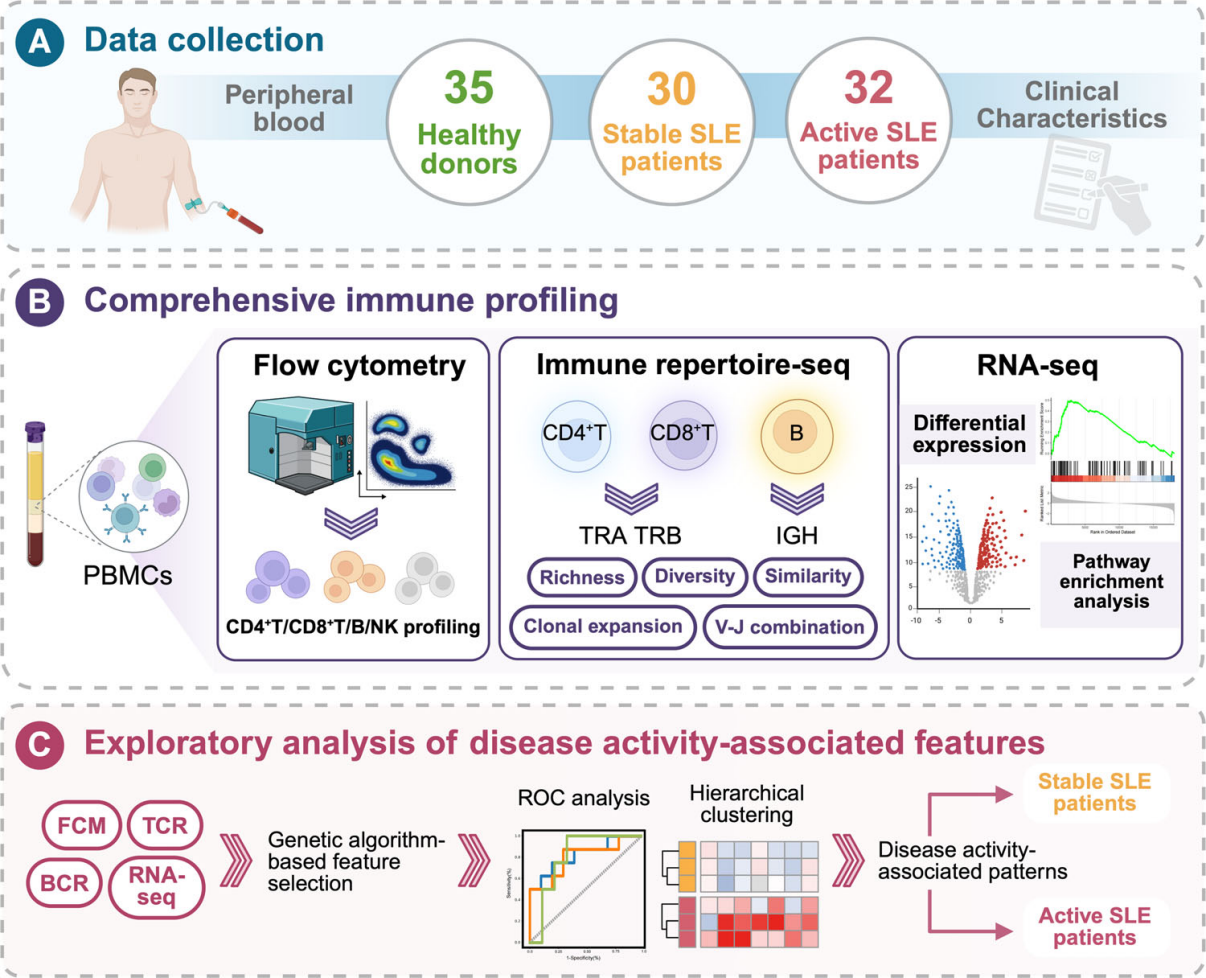
Immunoprofiling of peripheral blood of SLE patients and HDs. To characterize systemic immune features associated with disease activity, peripheral blood was collected from 32 active SLE patients, 30 stable SLE patients, and 35 healthy donors. Comprehensive immune profiling was performed using flow cytometry, immune repertoire sequencing, and bulk RNA sequencing. Finally, label‐guided feature selection followed by hierarchical clustering was used to explore multi‐omics patterns associated with SLE disease activity.

**TABLE 1:** Clinical characteristics of SLE patients and healthy donors.

	Stable SLE patients (*n* = 30)	Active SLE patients (*n* = 32)	Healthy donors (*n* = 35)
**Gender**			
Male	2 (6.67%)	6 (18.75%)	6 (17.14%)
Female	28 (93.33%)	26 (81.25%)	29 (82.86%)
**Age, years**	35 (28.75, 44.25)	43 (32.50, 54.75)	35 (28.50, 40.50)
**Disease duration, months**	60 (21.00, 95.00)	24 (1.00, 77.75)	−
**SLEDAI**	2 (0.00, 4.00)	10 (5.50, 18.50)	−
**Clinical characteristics**			
Nervous system involvement	0 (0%)	0 (0%)	−
Vasculitis	0 (0%)	0 (0%)	−
Arthritis	0 (0%)	10 (31.25%)	−
Myositis	0 (0%)	1 (3.13%)	−
Hematuria	4 (13.33%)	13 (40.63%)	−
Proteinuria	6 (20.00%)	18 (56.25%)	−
Rash	0 (0%)	13 (40.63%)	−
Alopecia	0 (0%)	7 (21.88%)	−
Oral ulcer	0 (0%)	5 (15.63%)	−
Pleurisy	0 (0%)	0 (0%)	−
Pericarditis	0 (0%)	0 (0%)	−
Fever	0 (0%)	7 (21.88%)	−
Thrombocytopenia	0 (0%)	6 (18.75%)	−
**Laboratory results**			
Anti‐dsDNA antibody, IU/mL	223.0 (72.20, 354.40)	536.8 (112.88, 910.63)	−
C3, mg/dL	103 (78.50, 120.50)	55 (39.00, 94.25)	−
C4, mg/dL	20 (12.00, 27.00)	7 (2.50, 13.00)	−
WBC, ×10^9^/L	5.60 (4.74, 7.20)	5.24 (4.03, 6.88)	−
Hemoglobin, g/L	130 (120.50, 138.50)	109 (96.75, 123.75)	−
Platelet, ×10^9^/L	239 (163.50, 287.50)	206 (117.50, 264.00)	−
ESR, mm/h	12 (7.00, 19.50)	33 (17.00, 46.75)	−
CRP, mg/L	0.70 (0.43, 3.40)	4.15 (1.00, 14.25)	−

*Note*: Continuous variables are presented as median (Q1, Q3), and categorical variables as *n* (%).

Abbreviations: anti‐dsDNA antibody: anti‐double‐stranded DNA antibody; C3: complement component 3; C4: complement component 4; CRP: C‐reactive protein; ESR: erythrocyte sedimentation rate; SLEDAI: systemic lupus erythematosus disease activity index; WBC: white blood cell.

**TABLE 2:** Background treatments at time of sampling.

Treatment category	Stable SLE patients (*n* = 30)	Active SLE patients (*n* = 32)
**Glucocorticoid dose, mg/day**	5 (0.0, 7.5)	5 (0.0, 10.0)
**Hydroxychloroquine, HCQ**	21 (70.0%)	18 (56.3%)
**Conventional immunosuppressants**		
Mycophenolate mofetil, MMF	6 (20.0%)	5 (15.6%)
Leflunomide, LEF	1 (3.3%)	0 (0.0%)
Tacrolimus, TAC	4 (13.3%)	2 (6.3%)
Cyclosporine A, CsA	0 (0.0%)	1 (3.1%)
Cyclophosphamide, CYC	0 (0.0%)	0 (0.0%)
**Biologics**		
Rituximab, RTX	1 (3.3%)	0 (0.0%)
Belimumab, BEL	1 (3.3%)	1 (3.1%)

*Note*: Glucocorticoid dose is presented as median (Q1, Q3); categorical variables are presented as *n* (%).

FCM demonstrated distinct immune dysregulation in SLE patients, notably marked by decreased CD4+ T and NK cells, increased CD8+ T cells, and an exhausted phenotypic shift from naïve toward effector/memory subsets in T cell compartments (Figure S1).

### 2.2 Analysis of TCR β Chain (TRB) and *α* Chain (TRA) Repertoire in CD8+ T Cells From SLE Patients

While TRB is commonly used in bulk TCR repertoire studies as a clonotype proxy because of its high CDR3β junctional diversity, T‐cell antigen recognition is determined by both TRA and TRB chains. Using RNA‐based 5’RACE amplification with TRAC‐ and TRBC‐specific reverse primers, we profiled TRA and TRB repertoires in sorted T‐cell fractions, enabling a more comprehensive assessment of chain‐specific TCR remodeling in SLE.

To investigate the TCR immune repertoire, we leveraged a multifaceted approach, including representative metrics revealing clonotype richness (i.e., clonotype numbers), diversity (e.g., normalized Shannon's entropy, Inverse Simpson index, DE50 value, Gini coefficient), clonal expansion (e.g., Top 1–100 most abundant clones), measures of inter‐repertoire similarity (e.g., Morisita index and Jaccard index), and analysis of V‐J gene usage biases to reveal group's heterogeneity in antigen‐driven selection (see Section 4 “TCR and BCR data analysis”).

CD8+ TRB clonotype richness, measured by normalized clonotype numbers (Figure 2A), was much lower in SLE. Patients with SLE also exhibited lower normalized Shannon's entropy and DE50 values and higher Gini coefficients (Figure 2B–D, see Section 4), indicating SLE patients had lower clonal diversity of CD8+ TRB.

**FIGURE 2:**
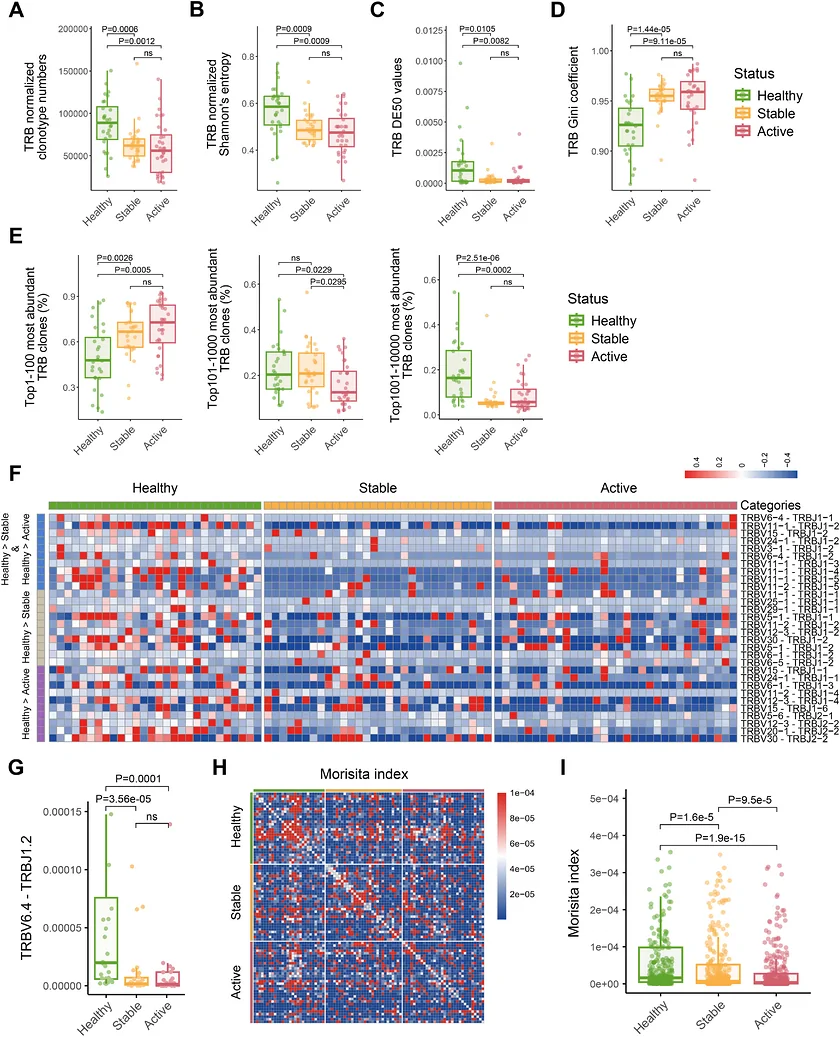
TCR β chain repertoire of CD8+ T cells in the peripheral blood of SLE patients and HDs. (A) The number of unique CD8+ TRB clones, a measure reflecting clonotype richness, among HDs, stable and active SLE patients. (B) Box plot of CD8+ TRB normalized Shannon's entropy, a measure reflecting clonotype diversity, among HDs, stable and active SLE patients. (C) Box plot of CD8+ TRB DE50, a measure reflecting clonotype diversity, among HDs, stable and active SLE patients. (D) Box plot of CD8+ TRB Gini coefficient, a measure reflecting clonotype diversity, among HDs, stable and active SLE patients. (E) Proportion of the top 1–100, 101–1000, and 1001–10,000 most abundant CD8+ TRB clones. (F) Heatmap illustrating the CD8**+** TRB V‐J gene combination usage. Rows represent different V‐J combinations, and columns represent individual patients or HDs. (G) Relative frequency of the TRBV6.4_TRBJ1.2 combination across groups. (H) Heatmap of pairwise CD8+ TRB repertoire similarity measured by the Morisita index. Rows and columns represent individual participants, grouped as HDs, stable, and active SLE patients. Colors indicate Morisita index values, with higher values representing greater repertoire similarity. (I) The overlap of shared CD8+ TRB clones is illustrated by Morisita index. Note: ns, not significant; HDs, healthy donors. Each dot represents one individual sample. Box plots show the median and interquartile range. *n* = 28 HDs, 30 stable SLE patients, and 32 active SLE patients.

The proportions of the top 1–100 clones, top 101–1000 clones, and top 1001–10,000 clones in TRB were calculated to illustrate clonal expansion. The top 1–100 TRB clones exhibited a higher percentage in SLE patients, especially in patients with active SLE (*p* = 0.0005) (Figure 2E). These results indicated higher TRB clonal expansion in SLE patients than in HDs. Similar results for CD8+ TRA are presented in Figure S2A–D.

To gain a more comprehensive understanding of the TCR repertoire, we profiled V‐J combinations. Several V‐J combinations were significantly more prevalent in HDs than in SLE patients, whereas no V‐J combinations showed significant enrichment in SLE, suggesting a more heterogeneous, low‐frequency distribution of V‐J combinations in SLE (Figure 2F,G). The pattern of CD8+TRA V‐J combination usage was similar to that of TRB (Figure S2E,F).

While metrics assessing TCR repertoire did not show significant differences between stable and active SLE patients in CD8+T cells, there was a trend toward lower diversity and higher clonal expansion in active patients compared with stable ones. Nevertheless, a statistically decreasing trend across the groups of HDs, stable and active SLE patients existed in the analysis of TCR similarity, using both the Morisita and Jaccard indexes (see Section 4) (Figure 2H,I; Figure S2G,H). This finding suggested that SLE patients lack the specific TRB and TRA similarity patterns found in HDs, and that the extent of this loss of repertoire similarity is associated with disease activity.

### 2.3 Analysis of TRA and TRB Repertoire in CD4+ T Cells From SLE Patients

By applying the same analytical framework used for CD8+T cells, we next extended our immunoprofiling approach to the CD4+T cell compartment. Consistent with the patterns observed in CD8+TCR repertoires, the number of unique CD4+TRA and TRB clones was significantly reduced in both stable and active SLE patients compared with HDs, and the Inverse‐Simpson index, normalized Shannon's entropy, and Gini coefficient were all impaired in SLE patients’ CD4+T cells (Figure 3A,B). The top 1–100 CD4+TRA and TRB clones accounted for a higher proportion in SLE patients (Figure 3C,D), indicating higher clonal expansion in SLE patients. Multiple V‐J combinations were more prevalent in HDs (Figure 3E–H), indicating that V‐J combinations in SLE patients may have higher heterogeneity. Overall, CD4+TRA/TRB repertoires showed parallel alterations in richness, diversity, clonal expansion, and V‐J usage biases to those observed in CD8+T cells, reinforcing the consistency of TCR repertoire changes in SLE.

**FIGURE 3:**
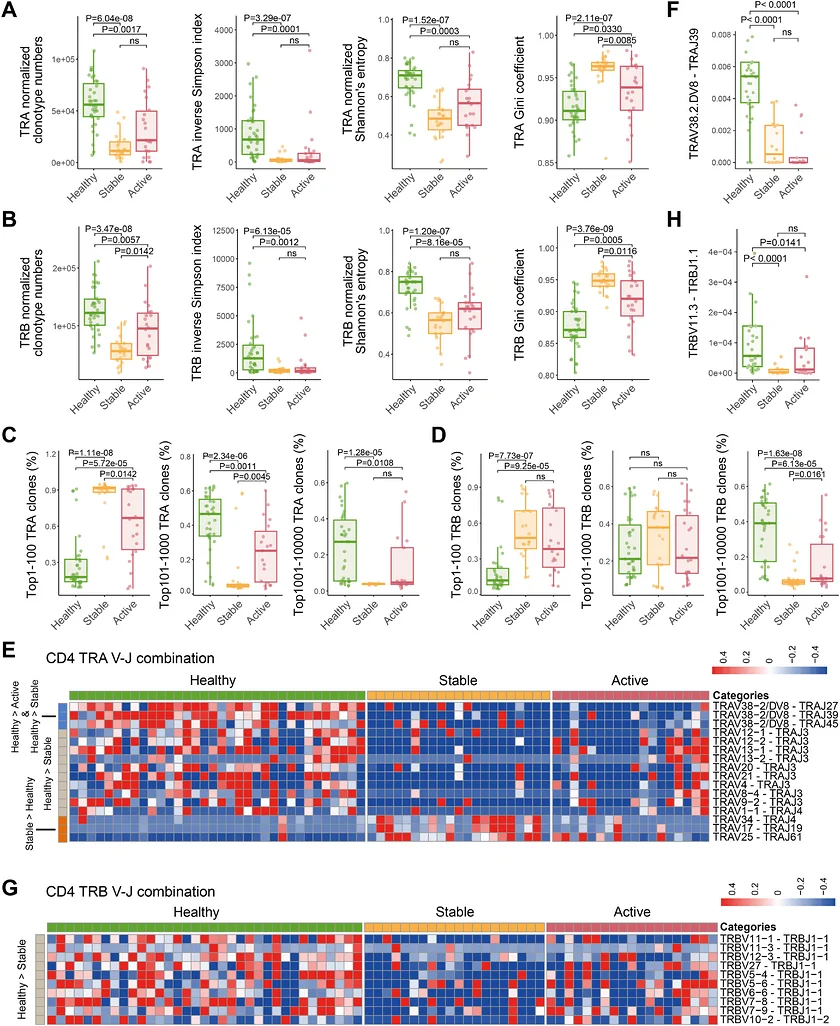
TCR α and β chain repertoires of CD4+ T cells in the peripheral blood of SLE patients and HDs. (A) The number of unique CD4+ TRA clones, the Inverse‐Simpson index, normalized Shannon's entropy, and Gini coefficient among HDs, stable and active SLE patients. (B) The number of unique CD4+ TRB clones, the Inverse‐Simpson index, normalized Shannon's entropy, and Gini coefficient among HDs, stable and active SLE patients. (C) Proportion of the top 1–100, 101–1000, 1001–10,000 most abundant CD4+ TRA clones. (D) Proportion of the top 1–100, 101–1000, and 1001–10,000 most abundant CD4+ TRB clones. (E) Heatmap illustrating the CD4**+** TRA V‐J gene combination usage. Rows represent different V‐J combinations, and columns represent individual patients. (F) Relative frequency of the TRAV38.2.DV8‐TRAJ39 combination across groups. (G) Heatmap illustrating the CD4**+** TRB V‐J gene combination usage. Rows represent different V‐J combinations, and columns represent individual patients. (H) Relative frequency of the TRBV11.3‐TRBJ1.1 combination across groups. Note: ns, not significant; HDs, healthy donors. Each dot represents one individual sample. Box plots show the median and interquartile range. *n* = 35 HDs, 22 stable SLE patients, and 22 active SLE patients.

We further investigated CD4+TCR differences between active and stable SLE patients, in which active SLE patients showed a tendency toward lower clonality and higher diversity compared with stable SLE patients (Figure 3A–D). This pattern may reflect altered CD4+ T‐cell subset composition during active disease, as redistribution or expansion of naïve, activated, memory, and B‐cell‐helper populations could reshape the bulk CD4+ TCR profile. Consistently, our flow cytometry data showed an increased frequency of naïve CD4+ T cells in active patients (Figure S1B), which may partly contribute to higher bulk repertoire diversity. As for clone similarity, consistent with CD8+ TCR, CD4+ TRB also exhibited a stepwise decrease among HDs, stable and active SLE patients (Figure S3A), but CD4+TRA clone similarity increased in active patients (Figure S3B).

### 2.4 Transcriptional Features of Peripheral CD8+ T Cells of SLE Patients and HDs

CD8+ T cells are increasingly implicated in SLE activity and tissue injury [27, 28, 29, 30], making them a relevant compartment for transcriptomic analysis. Based on our above TCR results, the CD8+TCR repertoire also showed greater consistency in the stepwise changes among HDs and stable and active SLE patients in terms of clonotype richness, diversity, V‐J usage and similarity. To determine if this shifted repertoire was linked to a parallel shift in the transcriptional landscape, we profiled the transcriptomes of CD8+T cells.

In this study, CD8+T cells were elevated in active SLE patients compared with HDs (Figure S1A). Transcriptomic analysis revealed that the numbers of differentially expressed genes (DEGs) in the active versus healthy and stable versus healthy comparisons were both higher than those observed in the active versus stable comparison (Figure 4A–C), with substantial DEG overlap between SLE groups (Figure S4A, Table S1). Genes related to effector function, exhaustion, cytotoxicity, TCR signaling pathway, and IFN response are shown in Figure S4B–D. Compared with stable SLE patients, active SLE patients showed upregulation of genes related to T‐cell activation and TCR‐associated signaling (Figure S4D)—including the AP‐1 complex components (*FOS*, *FOSB*, *FOSL1*, *BATF*), the calcium‐regulated transcription factor *NFATC4*, and the calcium sensors (*S100A6*, *S100A11*). These genes collectively form a critical module that translates TCR engagement into transcriptional activation, clonal expansion, and effector differentiation. These transcriptional changes are consistent with enhanced CD8+ T‐cell activation and may be associated with the repertoire‐level tendency toward increased clonality and reduced diversity observed in active disease. Several representative genes, including *CD38*, *S100A11*, *IFITM3*, and *LAG3*, showed a stepwise increase from HDs to stable and active SLE patients (Figure 4D–G), consistent with progressive CD8+ T‐cell activation, interferon‐associated responses, checkpoint upregulation, and inflammatory dysregulation across disease states.

**FIGURE 4:**
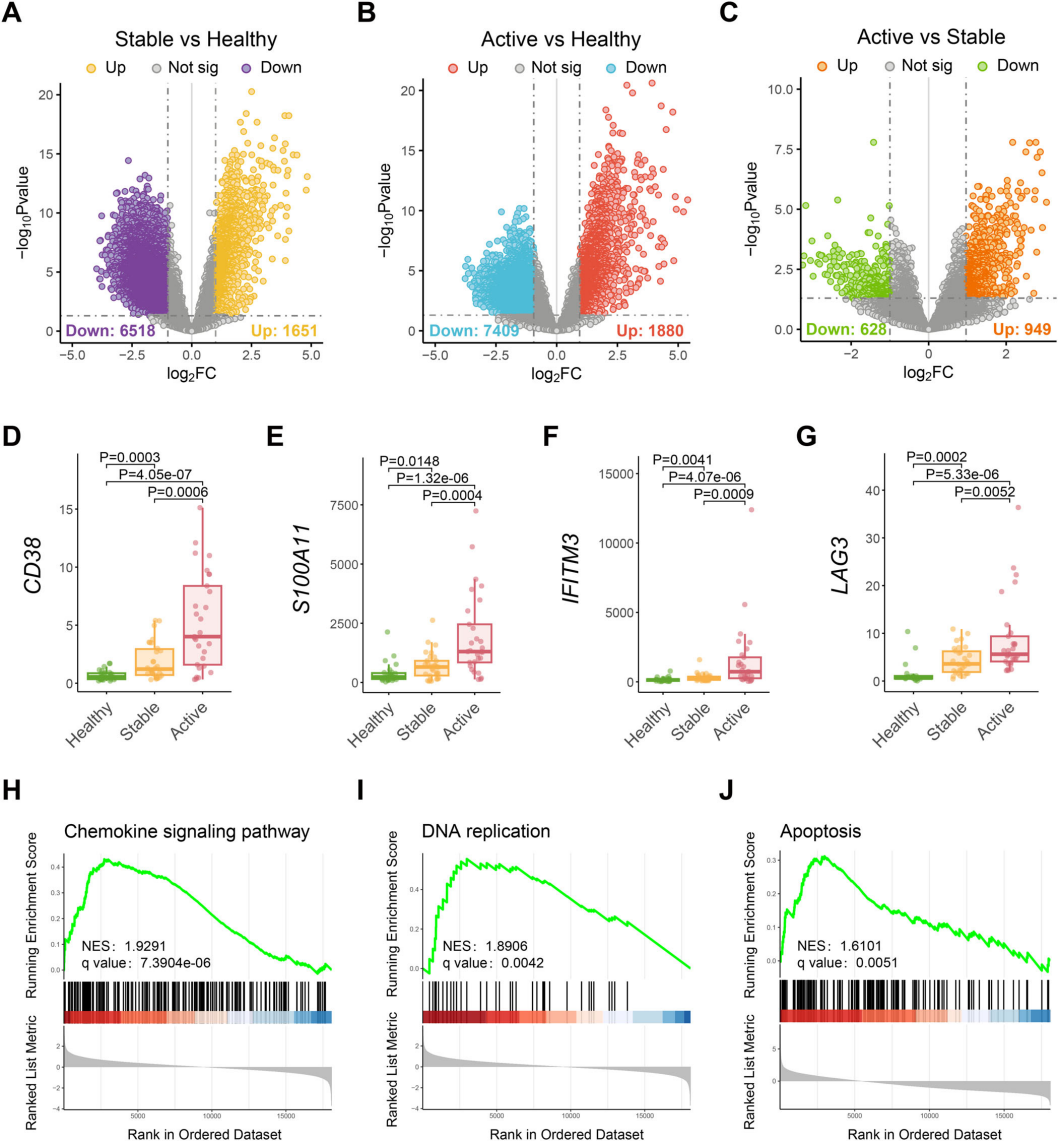
Transcriptional features of peripheral CD8+ T cells of SLE patients and HDs. (A) Volcano plot displaying DEGs in peripheral CD8+ T cells between stable SLE patients and HDs. (B) Volcano plot displaying DEGs in peripheral CD8+ T cells between active SLE patients and HDs. (C) Volcano plot displaying DEGs in peripheral CD8+ T cells between active and stable SLE patients. (D–G) The expression of *CD38*, *S100A11*, *IFITM3*, and *LAG3* across groups. (H–J) Pathway enrichment analysis of the chemokine signaling pathway, DNA replication, and apoptosis pathway between active and stable SLE patients. Note: ns, not significant; DEGs, differentially expressed genes; HDs, healthy donors. *n* = 28 HDs, 30 stable SLE patients, and 32 active SLE patients.

Pathway analysis showed upregulation of chemokine signaling, DNA replication, and apoptosis pathways in active versus stable SLE (Figure 4H–J), consistent with inflammatory signaling, proliferative activity, and cell death‐related transcriptional programs in active SLE CD8+ T cells.

### 2.5 Analysis of Immunoglobulin Heavy Chain (IGH) Repertoire in B Cells From SLE Patients

Given the central role of B cells in SLE, we analyzed the BCR IGH repertoire in SLE patients and HDs. We defined isotype‐specific IGH ratio as the proportion of read counts from a given immunoglobulin isotype relative to the total IGH read count within each sample. The IgA isotype ratio was elevated in SLE patients, whereas IgD, IgE, and IgM isotype ratios were lower in SLE patients (Figure 5A). IgG was notably higher in active SLE than in stable SLE and HDs, in line with the overproduction of autoantibodies in active SLE patients (Figure 5A).

**FIGURE 5:**
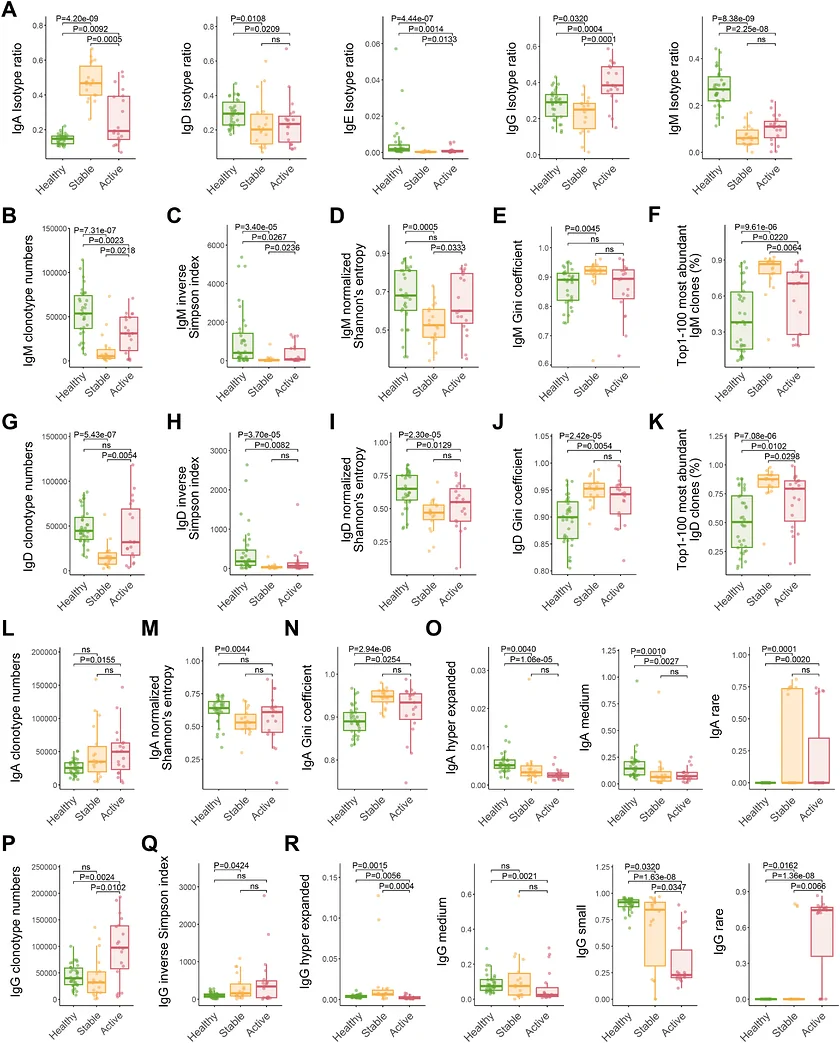
BCR immunoglobulin heavy chain repertoire for B cells in the peripheral blood of SLE patients and HDs. (A) IgA, IgD, IgE, IgG, and IgM isotype ratio among HDs, stable and active SLE patients. (B) The number of unique IgM BCR clones among HDs and stable and active SLE patients. (C‐E) The Inverse‐Simpson index, normalized Shannon's entropy, and Gini coefficient of IgM BCR, measures reflecting clonal diversity, among HDs, stable and active SLE patients. (F) Proportion of the top 1–100 most abundant IgM BCR clones. (G) The number of unique IgD BCR clones among HDs and stable and active SLE patients. (H–J) The Inverse‐Simpson index, normalized Shannon's entropy, and Gini coefficient of IgD BCR, measures reflecting clonal diversity, among HDs and stable and active SLE patients. (K) Proportion of the top 1–100 most abundant IgD BCR clones. (L) The number of unique IgA BCR clones among HDs and stable and active SLE patients. (M, N) The normalized Shannon's entropy and Gini coefficient of IgA BCR among HDs, stable and active SLE patients. (O) Distribution of IgA BCR clone sizes across groups. Clonotype size categories were defined according to isotype‐normalized clonal frequency as described in Section 4. (P) The number of unique IgG BCR clones among HDs, stable and active SLE patients. (Q) The Inverse‐Simpson index of IgG BCR among HDs, stable and active SLE patients. (R) Distribution of IgG BCR clone sizes across groups. Note: ns, not significant; HDs, healthy donors. Each dot represents one individual sample. Box plots show the median and interquartile range. *n* = 34 HDs, 18 stable SLE patients, and 19 active SLE patients.

As for BCR clone characteristics, IgM and IgD showed parallel changes with T cells of reduced richness (Figure 5B,G) and decreased diversity (Figure 5C–F,H–K) in SLE patients. In active SLE patients, there was a rebound of IgM clonotype richness as well as diversity toward HDs compared with stable patients (Figure 5B–F), likely reflecting broader IgM compartment remodeling responding to abundant self‐antigens in the active stage of SLE, thereby diluting pre‐existing dominant clones and diversifying IgM BCRs.

IgA and IgG repertoires exhibited distinct clonal characteristics: in SLE patients, IgA showed increased clonotype richness (Figure 5L), reduced evenness (Figure 5N) without significant changes in overall diversity (Figure 5M; Figure S5A–B) and decreased hyper‐expanded clones (Figure 5O; Figure S5C). IgG in SLE patients exhibited enhanced clone diversity accompanied by increased clonotype richness and enrichment of rare IgG clones (see Section 4) mainly in active SLE patients (Figure 5P–R; Figure S5D–G), suggesting that persistent stimulation by multiple autoantigens may contribute to a diversified repertoire without a single predominant specificity. Similar to TCR, specific IGH isotype V‐J combinations were more abundant in HDs (Figure S5H–O).

To account for potential age and sex imbalance across groups, we further performed rank‐based linear models with disease status as the main effect and age and sex as covariates for repertoire features. The FDR‐adjusted *p*‐values derived from age‐ and sex‐adjusted estimated marginal means were highly concordant with those from Mann–Whitney *U* tests, indicating that the major repertoire associations were not driven by age or sex imbalance (Table S2).

### 2.6 Correlation Between Immune Repertoire and Disease Activity of SLE Patients

We next performed receiver operating characteristic (ROC) analysis to evaluate the within‐cohort discriminatory patterns of immune repertoire and transcriptomic features in distinguishing active from stable SLE. CD4+ and CD8+ TCR‐derived features and the transcriptional signature of CD8+ T cells showed moderate discriminatory ability, with top 1–100 CD4+ TRA clones (%), top 101–1000 CD8+ TRB clones (%), and S100A11 yielding the highest AUC within their group (Figure 6A–C). Notably, BCR‐derived features, including IgG isotype ratio, IgG hyper‐expanded clones, and IgA isotype ratio, attained AUC values of 0.8684, 0.8450, and 0.8363, respectively (Figure 6D). These findings highlight BCR repertoire metrics, especially IgG‐ and IgA‐associated features, as candidate markers linked to disease activity in this cohort, together with TCR‐ and CD8 T‐cell RNA‐seq‐derived features.

**FIGURE 6:**
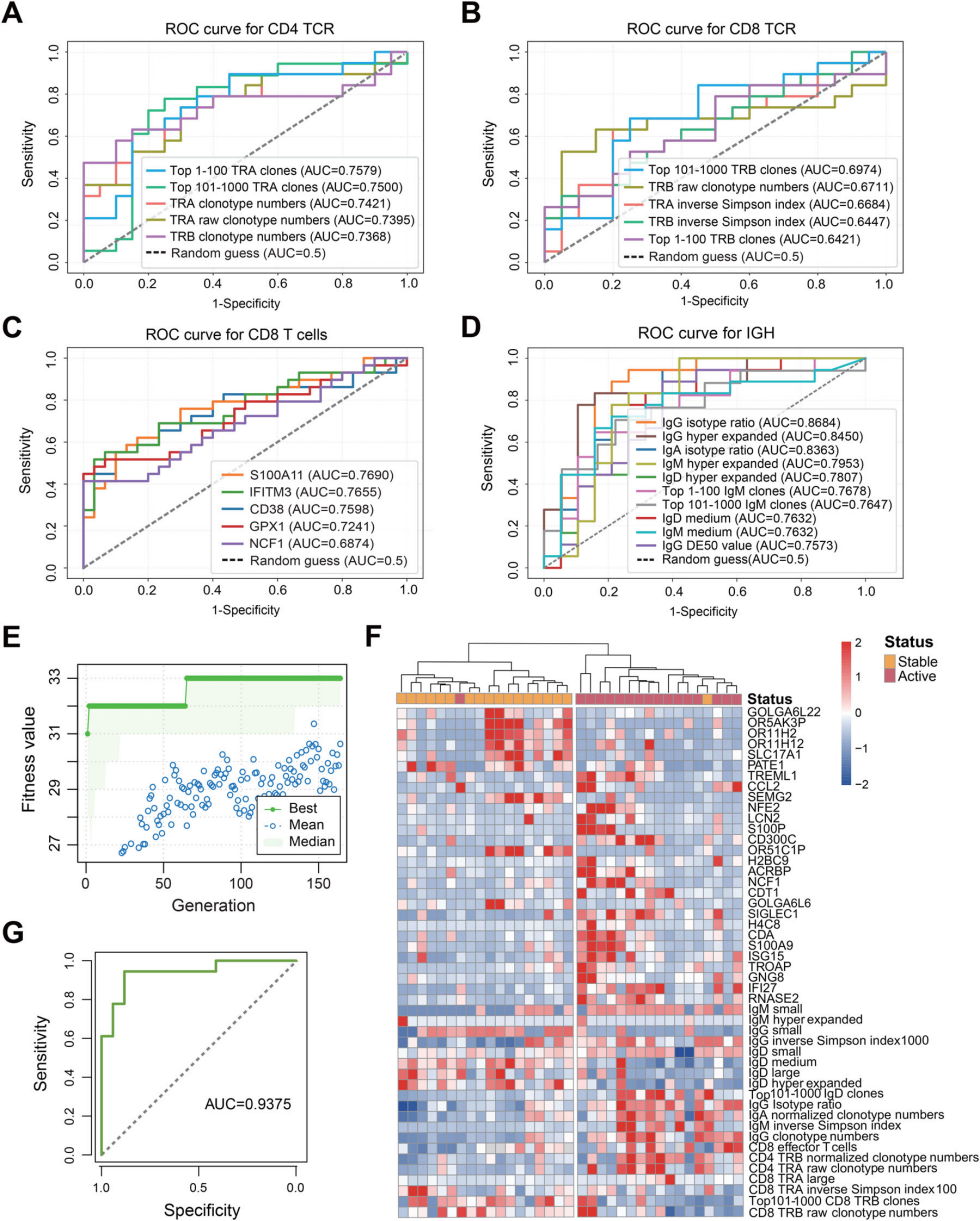
Exploratory analysis of disease‐activity‐associated immune features and integrative clustering in SLE. (A) ROC curve quantifying the degree of separation between stable and active SLE patients based on individual TCR features of CD4+ T cells. (B) ROC curve quantifying the degree of separation between stable and active SLE patients based on individual TCR features of CD8+ T cells. (C) ROC curve quantifying the degree of separation between stable and active SLE patients based on individual transcriptional features of CD8+ T cells. (D) ROC curve quantifying the degree of separation between stable and active SLE patients based on individual BCR IGH features. (E) Optimization of feature selection using a genetic algorithm. The best, mean, and median fitness values across generations are shown. (F) Heatmap of hierarchical clustering using the optimized feature subset. Columns represent individual patients (*n* = 35), and rows represent selected features (FACS, TCR, BCR, and RNA‐seq). (G) ROC curve descriptively summarizing the apparent within‐cohort separation of stable and active SLE patients based on the cluster‐derived centroid‐distance score.

Next, we conducted a genetic algorithm‐based label‐guided feature selection followed by hierarchical clustering. As shown in the optimization plot, the algorithm converged steadily, with a clear improvement in fitness values over generations (Figure 6E). As shown in Figure 6F, we included six features from TCR, 13 from BCR, 28 from RNA‐seq, and one from FACS (CD8+ Effector T). The heat map revealed clear group‐level separation between active and stable patients, with only one active and one stable sample assigned to the opposite cluster. To quantify the within‐cohort separation of the resulting clustering, a continuous discrimination score was calculated for each sample based on its relative distance to the cluster centroids. This score was used to compute a ROC curve. The resulting AUC was 0.9375, indicating apparent within‐cohort separation between active and stable cases (Figure 6G). Because disease‐activity labels contributed to feature screening and GA optimization in the same cohort, this AUC should be interpreted as an exploratory measure of within‐cohort separation rather than an unbiased estimate of out‐of‐sample predictive performance or generalizability. One‐way ANOVA showed that disease activity accounted for approximately 63% of the variance in this distance score (*η* ^2^ ≈ 0.63; *F* = 57.17, *p* = 1.07 × 10^−8^), showing a strong within‐cohort association between disease activity and the derived distance score.

In addition, systemic inflammation response index (SIRI) and systemic immune‐inflammation index (SII) are inflammation‐related clinical indices that have recently been associated with disease activity in SLE patients [32]. While both indices exhibited statistically significant discriminatory ability in our cohort (SIRI: AUC = 0.6586; SII: AUC = 0.6724; both *p* < 0.05) (Figure S6A,B), our multi‐omics profiling may provide complementary immune information that could be considered alongside these clinical indices when characterizing SLE disease activity.

To further assess the consistency of the identified TCR‐based signatures, we further analyzed an independent set of SLE patients using the remaining CD8+ TCR repertoires that were not included in the integrative multi‐omics analysis. This validation cohort consisted of 27 additional SLE patients for whom CD8+ TCR sequencing data were available but lacked complete multimodal measurements. Using the four CD8+ TCR‐derived parameters identified in the primary analysis, we generated a normalized heatmap to visualize repertoire patterns across active and stable patients (Figure S7). In this validation cohort, clustering based solely on the four CD8+ TCR‐derived parameters again partitioned the samples into two major groups, which broadly corresponded to stable and active disease states. This overall binary structure was consistent with the separation observed in the integrative multi‐omics analysis, despite the substantially reduced feature space. In contrast to the integrative analysis, which showed only two discordant assignments, clustering based solely on CD8+ TCR features resulted in an increased number of discordant samples (eight cases). This increase is likely attributable to the reduced feature space and the absence of complementary omics layers. This analysis provides independent validation of the TCR‐associated component of the findings, but does not constitute validation of the complete integrative multi‐omics pipeline.

## 3 Discussion

SLE pathogenesis involves profound dysregulation of both the adaptive and innate immune systems [3]. Although immune repertoire perturbations are increasingly recognized in autoimmunity, studies integrating TCR, BCR, and transcriptomic profiling in SLE remain limited. Here, we used a multimodal approach combining CD4+/CD8+ T‐cell repertoire sequencing, BCR IGH sequencing, and CD8+ T‐cell RNA‐seq to characterize peripheral immune remodeling in SLE and, more importantly, identified repertoire and transcriptomic features associated with SLE disease activity and explored their ability to separate active and stable SLE within the current cohort.

Using this integrated framework, we first characterized SLE‐associated immune repertoire remodeling in comparison with HDs. We applied a comprehensive set of repertoire metrics to delineate SLE‐specific immune repertoire alterations beyond conventional diversity‐based measurements. In both CD4+ and CD8+ TCR repertoires, including TRA and TRB chains, SLE patients demonstrate a systemic shift toward reduced richness, diversity, and similarity compared with HDs, along with more heterogeneous V‐J usage patterns. In BCR IGH repertoires, SLE patients showed isotype‐specific BCR repertoire remodeling. In the unswitched BCR repertoire, namely IgM and IgD compartments, the isotype ratio was contracted, accompanied by reduced clonotype richness and diversity. IgA BCR repertoires in SLE showed prominent isotype expansion and increased clonotype richness with reduced evenness and decreased hyper‐expanded clones, suggesting that IgA remodeling was driven by broad repertoire expansion with uneven clonal distribution rather than by the dominance of a few highly expanded clonotypes. IgG BCR repertoires were characterized by increased clonotype diversity in total SLE patients, with increased isotype ratio, clonotype richness, and enrichment of rare IgG clones mainly in active SLE patients, suggesting broad expansion and remodeling of the class‐switched IgG compartment to low‐frequency clones rather than expansion driven by a few dominant clones.

Having established these SLE‐specific repertoire abnormalities, we next asked whether peripheral immune features could further distinguish disease activity states within SLE. We therefore systematically evaluated the discriminatory performance of TCR‐derived, BCR‐derived, and CD8+ T‐cell transcriptomic features for separating active from stable SLE. BCR‐derived metrics, particularly IgG‐ and IgA‐associated features, exhibited marked within‐cohort discrimination between active and stable SLE.

This focus on activity‐associated immune signatures is consistent with previous peripheral blood transcriptomic studies in SLE. Recent studies have consistently demonstrated that peripheral blood RNA‐seq can successfully distinguish different SLE disease activity states, confirming its utility beyond merely separating patients from healthy donors. Whole‐blood transcriptomic studies reveal that active SLE is driven by upregulation of plasma cell, cell cycle, Toll‐like receptor (TLR), and inflammasome (e.g., *NLRP3*) pathways, whereas low disease activity states show recovery of lymphocyte populations and inhibitory immune checkpoints like *CTLA4* [33, 34, 35]. Longitudinal profiling of 27 immune cell subsets further showed disease activity as a major contributor to transcriptomic variation [28]. Furthermore, peripheral transcriptomes can effectively capture organ‐specific flares, such as active central nervous system (CNS) lupus [36] and lupus nephritis [37], underscoring their broad clinical relevance. Our study extends these foundational observations by directly assessing the potential of CD8 T‐cell transcriptomic features to stratify active versus stable SLE. Our work also assessed CD8 T‐cell transcriptomic features alongside TCR‐ and BCR‐derived repertoire metrics, extending prior transcriptomic observations to a multi‐omics framework for exploring immune patterns associated with disease activity.

Notably, within this cohort, BCR‐derived features, including IgG hyper‐expanded clones, IgG isotype ratio, and IgA isotype ratio, enabled separation between active and stable SLE. This likely reflects the central role of B‐cell activation in SLE flares, which are characterized by the rapid expansion of naïve‐derived activated effector B cells mainly through extrafollicular response [38, 39]. Such response is less dependent on classical germinal center (GC) selection and T follicular helper (Tfh) cell help, although they are not necessarily completely T cell‐independent, as T peripheral helper (Tph) cells may still provide IL‐21, CD40L, and other helper signals [40, 41]. However, as Tph cells represent only a minor fraction of the total CD4+ T‐cell pool [41], even during disease exacerbation, their specific TCR signatures may be significantly diluted by the vast background of other T cells. Consequently, BCR repertoire remodeling provides an amplified and direct imprint of ongoing humoral immune activation. These partially T cell‐dependent and T cell‐independent B‐cell activation pathways may also contribute to the asynchronous changes observed in our TCR and BCR data.

Among BCR‐associated features, IgA‐related metrics were of particular interest. Previous repertoire studies have reported marked IgA over‐representation in SLE, together with increased serum IgA levels, abnormal class‐switching patterns, and increased IgA CDR3 length [20]. Given the central role of IgA in mucosal immunity [42], these findings raise the possibility that mucosal immune responses or microbiome‐associated antigenic stimulation may represent an underappreciated driver of B‐cell activation in SLE. In line with these observations, our data showed prominent IgA repertoire remodeling in SLE. IgA isotype ratio also yielded an apparent AUC for distinguishing active from stable SLE within the current cohort. Although IgA‐associated features were not simply increased in active disease, their ability to distinguish active from stable SLE suggests that IgA remodeling captures a distinct layer of humoral immune dysregulation across disease states. These results extend the IgG‐centered framework of SLE flares and suggest that IgA‐associated repertoire remodeling may constitute an additional immune axis relevant to disease stratification.

The associations between global repertoire metrics and disease activity should also be interpreted in the context of marked interindividual repertoire heterogeneity in SLE. Although specific V‐J gene combinations and disease‐associated CDR3 sequences have shown strong performance in distinguishing SLE patients from HDs, their utility for stratifying disease activity within SLE is limited by profound interindividual repertoire heterogeneity. Previous studies have demonstrated that—despite statistical enrichment of certain clonotypes—expanded clones remain highly heterogeneous across SLE patients, with limited clonal overlap and no consistent shared CDR3 sequences or preferential V/J usage [12, 13]. Consistent with these observations, we found V‐J combination heterogeneity among patients in our cohort, indicating that active and stable SLE cannot be reliably separated by V‐J combination alone. Our analysis shifted toward global quantitative repertoire metrics. By evaluating parameters such as overall isotype ratio, clonotype richness, and diversity, we capture the structural immune remodeling associated with active disease, independent of individualized V‐J sequence backgrounds.

Finally, we integrated TCR, BCR, FACS, and transcriptomic features to explore whether complementary immune signals could jointly capture disease‐activity‐associated patterns. By applying a genetic algorithm for feature selection, we observed apparent within‐cohort separation. The resulting clusters broadly aligned with clinical disease activity states, with only one sample from each group assigned to the opposite cluster. Our findings suggest that multi‐omics integration with genetic algorithm‐based feature selection may help identify composite immune patterns associated with SLE disease activity. However, the limited sample size and high‐dimensional feature space may introduce overfitting, and the current analysis captures group‐level structure rather than individualized disease severity. Accordingly, the apparent in‐sample AUC is likely optimistic and should be regarded as an exploratory measure rather than an estimate of predictive performance. Independent multi‐omics cohorts or resampling procedures in which the complete feature‐selection pipeline is repeated within each training set will be required to assess generalizability.

Several other limitations should be acknowledged. This study was cross‐sectional and conducted at a single center, with all samples collected during a limited time window, and therefore the reported associations with disease activity should be interpreted as cross‐sectional rather than reflective of longitudinal progression or flare dynamics. Moreover, our analyses were restricted to peripheral blood and did not include tissue‐level data from affected organs, such as the kidney, or immune compartments in secondary lymphoid organs and bone marrow. This precludes direct linkage between circulating immune repertoire signatures and organ‐specific pathology, and limits our ability to assess long‐lived autoreactive lymphocyte populations that may contribute to SLE activity. Future studies will be required to determine how these repertoires and transcriptomic signatures relate to specific disease manifestations, as well as treatment responses and long‐term outcomes. In addition, upregulated CD8+ T‐cell genes identified by RNA‐seq were not further validated at the protein level by flow cytometry; therefore, future studies are needed to confirm whether these transcriptional changes are reflected in corresponding cellular phenotypes.

Overall, this study provides an integrated view of peripheral immune remodeling in SLE and highlights that combined TCR/BCR repertoire and transcriptomic profiling may help characterize disease activity‐associated immune patterns, with BCR IGH‐derived metrics showing marked within‐cohort associations with disease activity.

## 4 Materials and Methods

### 4.1 Study Population

During 2024, 32 active patients and 30 stable patients with SLE diagnosed according to the European League Against Rheumatism/American College of Rheumatology 2019 criteria from the Department of Rheumatology and Immunology at Ruijin Hospital were included in this study [43]. Patients with acquired immunodeficiency syndrome, cancer, or individuals who were less than 18 years old were not included in this study. The diagnosis of SLE was confirmed by two qualified rheumatologists. Demographic and clinical characteristics (e.g., fever, pericarditis, pleuritis, mouth ulcers, rash, arthritis, vasculitis, and nephritis), as well as laboratory tests (e.g., anti‐dsDNA IgG levels, ESR, WBC, Hb, PLT, CRP, C3, and C4), were collected from patients’ medical records. Thirty‐five HDs were included to evaluate the TCR and BCR in the general population. Systemic lupus erythematosus disease activity index (SLEDAI) scores were used to measure disease activity: 0–4: not active, 5–9: mildly active, 10–14: moderately active, >14: severely active. In our study, we defined patients with SLEDAI scores between 0 and 4 as having stable SLE, and those with scores of 5 or higher as having active SLE.

The study followed the ethical standards for human experimentation established in the Declaration of Helsinki and was approved by the Institutional Research Ethics Committee of Ruijin Hospital (ID: 2016–62), Shanghai, China. Informed consent was obtained from all participants in the study.

### 4.2 CD8+ T Cells and CD8− cells Sorting

PBMCs were isolated by Lymphoprep density gradient centrifugation. CD8+ T cells were positively selected using anti‐CD8 magnetic beads (BioLegend; MojoSort Human CD8 T cell isolation kit). Purified CD8+ T cells and the negative fraction (mainly CD4+ T cells and B cells) were immediately lysed for RNA extraction.

### 4.3 RNA Extraction and Sequencing

Extraction of RNA was performed per the manufacturer's protocol (Qiagen), with subsequent quantification achieved using a Qubit RNA BR assay kit (Thermo Fisher Scientific). Libraries for TCR sequencing were constructed from immune cells’ RNA. Furthermore, mRNA derived from CD8+ T cells and CD8‐T cells (primarily CD4+T cells and B cells) was subjected to reverse transcription for subsequent sequencing.

### 4.4 TCR and BCR Library Preparation and Sequencing

To precisely profile the adaptive immune repertoires, specific libraries were constructed based on the sorted cell fractions: the CD8+ T cell fraction was utilized for CD8+ TCR sequencing, while the corresponding CD8− fraction was subjected to CD4+ TCR sequencing and BCR IGH sequencing.

The amplification and sequencing of the immune repertoire were conducted using an optimized systemic platform based on a previously described method with modifications [44]. The procedure entailed a modified magnetic bead‐based cDNA purification method to improve the efficiency of immune repertoire enrichment and 5′RACE reverse transcription, followed by a two‐round polymerase chain reaction (PCR) amplification of the TCR and BCR variable regions. This amplification incorporated adapter‐containing reverse primers complementary to the TCR and BCR constant region (LeaImmucenter platform; LeaLing Bio). The resulting amplicons were then ligated to sequencing adaptors for paired‐end sequencing (150 bp) on the DNBSEQ‐T7 platform.

### 4.5 TCR and BCR Data Analysis

The generation of TCR and BCR repertoires was conducted by analyzing the paired‐end FASTQ sequencing files with a licensed copy of PyIR (v1.20.0). This tool functions as a high‐throughput wrapper for the Immunoglobulin Basic Local Alignment Search Tool (IgBLAST) and is characterized by its minimal software dependencies. Alignment and assembly processes were performed with the default settings. For downstream analyses, only sequences annotated as vj_in_frame = T and productive = T, and containing both CDR3 and FW4 sequences, were retained.

Repertoire metrics were assessed at three levels: repertoire richness, diversity/clonality, and clonal expansion. Repertoire richness was quantified by the clonotype number, defined as the total number of unique clonotypes detected in each sample.

Repertoire diversity and clonality were assessed using Shannon's diversity index, the Inverse Simpson index, DE50, and the Gini index. Shannon's diversity index was derived from its defining equation, wherein p*_i_* denotes the frequency of the *i‐*th clonotype in a sample possessing *N* distinct clonotypes.

$$H^{\prime }=-\sum_{i\;=1}^{N}p_{i}*\ln(p_{i}).$$

Normalized Shannon diversity entropy (NSDE) was:

$$NSDE=\frac{H^{\prime }}{\ln(N)}.$$

NSDE index is a score bounded between 0 (monoclonality) and 1 (maximum diversity) [45].

The Inverse Simpson index provides a measure of diversity, with higher values corresponding to greater repertoire diversity. The DE50 index represents the normalized rank at which cumulative clonotype frequency reaches 50%, also reflecting repertoire diversity. The Gini index measures clonal evenness on a scale from 0 (complete equality, indicative of diversity) to 1 (complete inequality, indicative of clonality).

Clonal expansions were also analyzed through two complementary classification systems. First, based on ranked relative abundance (“clonal space”), clones were grouped into the top 1–100, top 101–1000, and top 1001–10,000 for each sample, with the summed relative abundance of the top 1–100 clones defining the “top 1–100 clonal space”. Second, based on isotype‐ or chain‐normalized clonal frequency (Fi), defined as the proportion of counts for a given clone relative to the total counts of the corresponding receptor unit (TRA, TRB, or individual IGH isotypes) within each sample, clones were assigned to size categories: rare (Fi < 1 × 10^−6^), small (1 × 10^−6^ ≤ Fi < 1 × 10^−5^), medium (1 × 10^−5^ ≤ Fi < 1 × 10^−4^), large (1 × 10^−4^ ≤ Fi < 1 × 10^−3^), and hyper‐expanded (Fi ≥ 1×10^−3^).

### 4.6 TCR Repertoire Similarity Analysis

Convergence within the TCR repertoire was defined as the summed frequency of all clones that possessed a common variable gene and an identical CDR3 amino acid sequence. For similarity assessment of TCR clonotype composition and abundance between samples, the Morisita's overlap index was employed. The Morisita Index formula is:

$$M=\frac{2*\sum \left(Xi\;*\;Yi\right)}{\left[\left(\sum \left({Xi}^{2}\right)/\;X^{2}\right)+\left(\sum \left({Yi}^{2}\right)\;/\;Y^{2}\right)\right]*\;X*\;Y^{\prime }}$$

where Xi/Yi is the number of individuals of species I in sample X/Y; X/Y is the total number of individuals in sample X/Y.

The Jaccard index is used to measure the similarity of TCR clonotypes between two samples based on their shared and unique clonotypes.

The Jaccard index formula is:

$$J=\frac{|X\cap Y|\;}{|X\cup Y|},$$

where X/Y is the set of clonotypes present in sample X/Y; ∣X∩Y∣ is the number of clonotypes shared by both samples; ∣X∪Y∣ is the total number of distinct clonotypes across both samples.

### 4.7 CD8+ T Cells Bulk RNA‐seq Sequencing

Bulk RNA sequencing was performed on DNBSEQ‐T series platforms using the DNBSEQ‐T7RS High‐throughput Sequencing Kit (FCL PE150) V3.0 to generate 150 bp paired‐end reads. Reads were aligned to the GRCh38 reference genome using HISAT2, and transcripts were assembled with StringTie based on Ensembl annotation (release 108). Gene expression was quantified as transcripts per million (TPM). Count matrices were generated using featureCounts (v2.0.6) with the same annotation. For differential expression analysis, genes were retained if expressed (FPKM > 0) in at least 50% of samples in any biological condition. Analysis was performed using DESeq2 (v1.38.3). For CD8+ T cell clusters, gene set scores were computed by summing TPM values of constituent genes. Additionally, signature scores for curated gene sets were calculated by averaging log2(TPM+1) values of member genes, followed by scaling across both samples and gene sets.

### 4.8 Flow Cytometry Assay

Flow cytometry was used to identify cellular phenotypes in PBMCs. Commercially available monoclonal antibodies against lymphocytes and T cell subtypes were titrated to optimal concentrations. PBMCs were blocked, aliquoted, and stained with customized antibody panels. Specifically, PBMCs were centrifuged at 300×*g* for 5 min, resuspended, and treated with blocking buffer for 10 min at room temperature. Each sample was then aliquoted into 10 wells of a 96‐well plate, centrifuged again, and the supernatant was removed. Staining was performed using a custom antibody combination for 20 min at room temperature. After staining, cells were washed with 200 µL of FACS buffer, centrifuged, and the supernatant was removed. Data acquisition was performed on a Beckman CytoFLEX instrument.

### 4.9 Exploratory Label‐Guided Feature Selection and Clustering Analysis

We only analyzed samples for which CD8+ TCR, non‐CD8+ TCR, IGH repertoire, FACS, and RNA‐seq data were all available. This dataset included a cohort of 35 patients, comprising 18 stable and 17 active cases.

#### 4.9.1 Feature Selection

Features from immune repertoires and FACS were included if they showed an unadjusted *p*‐value < 0.1 in active vs. stable comparisons, yielding 56 features. RNA‐seq features were selected using FDR < 0.01 and |log_2_FC| > 2, yielding 64 genes. All selected features were combined into a 120‐feature matrix, with rows representing patient samples and columns representing scaled features. Because active/stable disease‐activity labels were used in these comparisons, this initial feature‐screening step was label‐guided.

#### 4.9.2 Genetic Algorithm‐Based Feature Selection

Because the number of features was large, we employed a genetic algorithm to identify an optimal subset of features that best separated active from stable samples via unsupervised clustering. The fitness function required: (1) two balanced clusters (≥12 samples each), and (2) high purity (≥70%) of clinical groups within clusters. Therefore, disease‐activity labels also contributed to the genetic algorithm‐based feature‐selection step, although the subsequent hierarchical clustering algorithm itself did not use clinical labels. The genetic algorithm used a population size of 200, a maximum of 200 iterations, a mutation rate of 0.2, and a crossover rate of 0.5, with early stopping after 100 nonimproving generations. The best fitness function value was 33, and we adopted the combination of the smallest number of features that could achieve this optimal value. We have made the genetic algorithm‐based feature selection code publicly available via a GitHub repository (https://github.com/dbtsshome/Genetic‐Algorithm‐based‐Feature‐Selection). The analysis repository includes executable R scripts, a README with input and execution instructions, example input data, package/version information, and representative expected outputs.

#### 4.9.3 Clustering Evaluation and Descriptive Within‐Cohort ROC Analysis

Hierarchical clustering (Ward's method, Euclidean distance) was applied to genetic algorithm‐selected features. Cluster assignment was compared with clinical labels. For ROC analysis, each sample's Euclidean distance to cluster centroids was calculated, with the distance difference used as a separation score. The ROC curve and AUC were computed using the pROC package to descriptively quantify the degree of separation between disease activity states rather than to evaluate a predictive model, and results were visualized in a heatmap.

### 4.10 Statistical Analysis

The significance threshold was adjusted for multiple testing via FDR correction when applicable, with details provided in the respective figure legends. For intergroup comparisons, the two‐tailed, nonparametric Mann–Whitney *U* test was applied. Rank‐based linear models were fitted for each feature in the CD8+ T cell repertoire and IGH repertoire with disease status as the main effect, adjusting for age and sex. Pairwise comparisons between disease states (healthy vs. stable, healthy vs. active, and stable vs. active) were performed using estimated marginal means derived from these age‐ and sex‐adjusted models, with Tukey correction applied for multiple comparisons using the emmeans R package. Correlations between variables were assessed using Spearman's rank correlation coefficient. The R statistical computing environment (version 4.4.1) was utilized for all analyses. Similarly, all graphical representations were generated using R scripts incorporating the “ggplot2” package and GraphPad PRISM software.

## Author Contributions

Y.D., C.Y., and J.Y. designed the study. J.W., J.T., D.Z., F.W., Y.Y., P.W., and H.L. were involved in sample preparation and processing. J.W., A.Y., X.L., and X.J. conducted statistical analyses and interpreted the results. J.W. and Z.Z. drafted the manuscript. J.Y. and Y.R. revised the manuscript. J.Y. has verified the underlying data. All authors have read and approved the final manuscript.

## Funding

This work was supported by the National Natural Science Foundation of China (82371798, 82672333), Fundamental Research Funds for the Central Universities (YG2025QNB44), Shanghai Rising‐Star Program (23QA1406200), Natural Science Foundation of Shanghai (23ZR1440500), Ruijin Hospital (2025TM002).

## Ethics Statement

The study was approved by the Institutional Research Ethics Committee of Ruijin Hospital (ID: 2016–62), Shanghai, China. Informed consent was obtained from all participants in the study.

## Conflicts of Interest

Wenbo Wang and Xiangjun Ji are employees of LeaLing Biopharma Co., Ltd. The remaining authors declare no conflicts of interest.

## Supporting information




**Figure S1**: Flow cytometry‐based evaluation of immune cells in peripheral blood of SLE patients and HDs.
**Figure S2**: TCR alpha chain repertoire of CD8^+^ T cells in the peripheral blood of SLE patients and HDs.
**Figure S3**: TCR alpha and beta chain repertoire of CD4^+^ T cells in the peripheral blood of SLE patients and HDs.
**Figure S4**: Transcriptional features of peripheral CD8^+^ T cells of SLE patients and HDs.
**Figure S5**: BCR heavy chain repertoire for B cells in the peripheral blood of SLE patients and HDs.
**Figure S6**: SLE disease activity discriminatory ability of systemic immune‐inflammation index (SII) and systemic inflammation response index (SIRI).
**Figure S7**: Heatmap of hierarchical clustering based on the CD8^+^ TCR‐derived feature subset.

**Table S1**: Differentially expressed genes identified by pairwise DESeq2 comparisons among active, stable, and healthy groups.

**Table S2**: Comparison of false discovery rate (FDR)adjusted *p* values obtained from age‐ and sex‐adjusted estimated marginal means (EMMs) and MannWhitney U tests for repertoire features.

## Data Availability

All sequencing data will be available upon reasonable request. Genetic algorithm–based feature selection code has been made publicly available via a GitHub repository (https://github.com/dbtsshome/Genetic‐Algorithm‐based‐Feature‐Selection). The analysis repository includes executable R scripts, a README with input and execution instructions, example input data, package/version information, and representative expected outputs.
