# Supplementary material for: Comprehensive Immune Repertoires of T Cell Receptor and B Cell Receptor Identify Key Immune Features Associated With Disease Activity of Systemic Lupus Erythematosus
Source: MedComm (2020). 2026 Oct 23;7(11):e71087. doi: 10.1002/mco2.71087 (PMC13693921; doi:10.1002/mco2.71087)
Supplement: Supplementary file 1 [file MCO2-7-e71087-s003.docx]

**Comprehensive immune repertoires of T cell receptor and B cell receptor identify key immune features associated with disease activity of systemic lupus erythematosus**

Jingyi Wu^1 #^, Zhuochao Zhou^1 #^, Anrui Yu^1 #^, Xia Lin^2 #^, Yuyang Ren^1^, Jialin Teng^1^, Dehao Zhu^3^, Fan Wang^3^, Yijun You^4^, Xiangjun Ji^5,6^, Puyu Wang^7^, Hongpu Li^7^, Chengde Yang^1 *^, Wenbo Wang^5,6 *^, Yawei Du^8 *^, Junna Ye^1 *^

1 Department of Rheumatology and Immunology, Ruijin Hospital, Shanghai Jiao Tong University School of Medicine, Shanghai, China

2 Department of Paediatrics, Ruijin Hospital, Shanghai Jiao Tong University School of Medicine, Shanghai, China

3 Department of Critical Care Medicine, Ruijin Hospital, Shanghai Jiao Tong University School of Medicine, Shanghai, China

4 Department of Infectious Diseases, Ruijin Hospital, Shanghai Jiao Tong University School of Medicine, Shanghai, China

5 Department of Oncology, Shanghai Tenth People’s Hospital, Tongji University School of Medicine, Shanghai

6 LeaLing Biopharma Co.,Ltd., Suzhou, China

7 Department of Rheumatology and Immunology, The Fourth Affiliated Hospital of Xinjiang Medical University, Xinjiang, China

8 Department of Orthopaedics, Shanghai Key Laboratory for Prevention and Treatment of Bone and Joint Diseases, Shanghai Institute of Traumatology and Orthopaedics, Ruijin Hospital, Shanghai Jiao Tong University School of Medicine, Shanghai, China

\# These authors contributed equally to this article.

* Correspondence to:

**Junna Ye**, MD & PhD

Department of Rheumatology and Immunology, Ruijin Hospital, Shanghai Jiao Tong University School of Medicine,

No. 197 Ruijin Second Road, Huangpu District, Shanghai 200025, China.

E-mail: yjn11981@rjh.com.cn

**Yawei Du,** PhD

Department of Orthopaedics, Shanghai Key Laboratory for Prevention and Treatment of Bone and Joint Diseases, Shanghai Institute of Traumatology and Orthopaedics, Ruijin Hospital, Shanghai Jiao Tong University School of Medicine,

No. 197 Ruijin Second Road, Huangpu District, Shanghai 200025, China.

E-mail: yaweidu@sjtu.edu.cn

**Wenbo Wang,** PhD

Department of Oncology, Shanghai Tenth People's Hospital, Tongji University School of Medicine, Shanghai; LeaLing Biopharma Co.,Ltd., Suzhou, China

No. 197 Ruijin Second Road, Huangpu District, Shanghai 200025, China.

E-mail: wbwang@llbiopharma.com

**Chengde Yang,** MD & PhD

Department of Rheumatology and Immunology, Ruijin Hospital, Shanghai Jiao Tong University School of Medicine,

No. 197 Ruijin Second Road, Huangpu District, Shanghai 200025, China.

E-mail: yangchengde@sina.com

**Figure S1. Flow cytometry-based evaluation of immune cells in peripheral blood of SLE patients and HDs.**

(A) Percentage of immune cells (T/CD4+ T/CD8+ T/B/NK cells) among HDs, stable and active SLE patients.
(B) Percentage of different CD4+ T cell clusters (naïve T, central memory T, effector memory T, effector T) among HDs, stable and active SLE patients.
(C) Percentage of different CD8+ T cell clusters (naïve T, central memory T, effector memory T, effector T) among HDs, stable and active SLE patients.

Footnotes: ns, not significant; HDs, healthy donors.

**Figure S2. TCR alpha chain repertoire of CD8+ T cells in the peripheral blood of SLE patients and HDs.**

(A) The number of unique CD8+ TRA clones among HDs, stable and active SLE patients.

(B-C) Box plot of CD8+ TRA Normalized Shannon’s entropy and Gini coefficient among HDs, stable and active SLE patients.

(D) Proportion of the top 1-100, 101-1000, 1001-10000 most abundant CD8+ TRA clones.

(E) Heatmap illustrating the CD8**+** TRA V-J gene combination usage. Rows represent different V-J combinations, and columns represent individual patients.

(F) Relative frequency of the TRAV12.1-TRAJ4 combination across groups.

(G-H) The similarity of CD8+ TRA clonotypes across samples assessed by Morisita and Jaccard indices.

Footnotes: ns, not significant; TCR, T cell receptor; HDs, healthy donors. Each dot represents one individual sample. Box plots show the median and interquartile range. n = 28 HDs, 30 stable SLE patients and 32 active SLE patients.

**Figure S3. TCR alpha and beta chain repertoire of CD4+ T cells in the peripheral blood of SLE patients and HDs.**

(A) Similarity of CD4+ TRB clonotypes across samples assessed by the Morisita index.

(B) Similarity of CD4+ TRA clonotypes across samples assessed by the Morisita index.

**Figure S4. Transcriptional features of peripheral CD8+ T cells of SLE patients and HDs.**

(A) Venn diagrams displaying DEGs identified from pairwise comparisons among HDs, stable SLE patients, and active SLE patients based on peripheral blood CD8^+^ T-cell bulk RNA-seq data.
(B) Volcano plot highlighting DEGs associated with CD8+ T cell activation/effector, exhaustion, cytotoxicity, TCR signaling and IFN response functions between HDs and stable SLE patients.
(C) Volcano plot highlighting DEGs associated with CD8+ T cell activation/effector, exhaustion, cytotoxicity, TCR signaling and IFN response functions between HDs and active SLE patients.
(D) Volcano plot highlighting DEGs associated with CD8+ T cell activation/effector, exhaustion, cytotoxicity, TCR signaling and IFN response functions between stable and active SLE patients.

Footnotes: HDs, healthy donors; TCR, T cell receptor; IFN, interferon; DEGs, differentially expressed genes.

**Figure S5. BCR heavy chain repertoire for B cells in the peripheral blood of SLE patients and HDs.**

(A) The Inverse-Simpson index of IgA BCR among HDs, stable and active SLE patients.

(B) Proportion of the top 1-100, 101-1000, 1001-10000 most abundant IgA BCR clones.

(C) Distribution of IgA BCR clone sizes across groups.

(D-E) The normalized Shannon’s entropy and Gini coefficient of IgG BCR among HDs, stable and active SLE patients.

(F) Proportion of the top 1-100, 101-1000, 1001-10000 most abundant IgG BCR clones.

(G) Distribution of IgG BCR clone sizes across groups..

(H) Heatmap illustrating the IgA BCR V-J gene combination usage. Rows represent different V-J combinations, and columns represent individual patients.

(I) Relative frequency of the IGHV3.23-IGHJ1 combination across groups.

(J) Heatmap illustrating the IgD BCR V-J gene combination usage.

(K) Relative frequency of the IGHV3.66-IGHJ4 combination across groups.

(L) Heatmap illustrating the IgG BCR V-J gene combination usage.

(M) Relative frequency of the IGHV6.1-IGHJ6 combination across groups.

(N) Heatmap illustrating the IgM BCR V-J gene combination usage.

(O) Relative frequency of the IGHV3.30-IGHJ5 combination across groups.

Footnotes: ns, not significant; BCR, B cell receptor; HDs, healthy donors. Each dot represents one individual sample. Box plots show the median and interquartile range. n = 34 HDs, 18 stable SLE patients and 19 active SLE patients.

**Figure S6. SLE disease activity discriminatory ability of systemic immune-inflammation index (SII) and systemic inflammation response index (SIRI).** Receiver operating characteristic (ROC) curves showing the SLE disease activity separation performance of SII (A) and SIRI (B). The area under the curve (AUC) and corresponding p value are shown in each panel. The red diagonal line represents the reference line for no discriminative ability.

**Figure S7. Heatmap of hierarchical clustering based on the CD8+ TCR-derived feature subset.** Rows represent CD8+ TCR repertoires from an independent SLE cohort that was not included in the integrative multi-omics analysis (n = 27), and columns represent the four CD8+ TCR-derived parameters identified in the primary analysis.
